# Few-Layer Graphene Sheet-Passivated Porous Silicon Toward Excellent Electrochemical Double-Layer Supercapacitor Electrode

**DOI:** 10.1186/s11671-018-2646-7

**Published:** 2018-08-17

**Authors:** Te-Hui Wu, Chih-Tse Chang, Chun-Chieh Wang, Shaikh Parwaiz, Chih-Chung Lai, Yu-Ze Chen, Shih-Yuan Lu, Yu-Lun Chueh

**Affiliations:** 1 Department of Materials Science and Engineering, National Tsing Hua University, Hsinchu, 30013 Taiwan; 2 Department of Chemical Engineering, National Tsing Hua University, Hsinchu, 30013 Taiwan; 3 Department of Physics, National Sun Yat-Sen University, Kaohsiung, 80424 Taiwan; 4 Frontier Research Center on Fundamental and Applied Sciences of Matters, National Tsing Hua University, Hsinchu, 30013 Taiwan

**Keywords:** Porous silicon, Graphene passivation, Supercapacitor, Areal capacitance, Hierarchical porous electrode

## Abstract

Few-layer graphene sheet-passivated porous silicon (PSi) as an outstanding electrochemical double-layer supercapacitor electrode was demonstrated. The PSi matrix was formed by electrochemical etching of a doped silicon wafer and was further surface-passivated with few-layer graphene sheets by a Ni-assisted chemical vapor deposition process where a wide range of porous PSi structures, including mesoporous, macroporous, and hybrid porous structures were created during the graphene growth as temperature increases. The microstructural and graphene-passivation effects on the capacitive performance of the PSi were investigated in detail. The hybrid porous PSi electrode, optimized in terms of capacitive performances, achieves a high areal capacitance of 6.21 mF/cm^2^ at an ultra-high scan rate of 1000 mV/s and an unusual progressing cyclic stability of 131% at 10,000 cycles. Besides mesopores and macropores, micropores were introduced onto the surfaces of the passivating few-layer graphene sheets with a KOH activation process to further increase the functioning surface area of the hierarchical porous PSi electrode, leading to a boost in the areal capacitance by 31.4% up to 8.16 mF/cm^2^. The present designed hierarchical porous PSi-based supercapacitor proves to be a robust energy storage device for microelectronic applications that require stable high rate capability.

**Electronic supplementary material:**

The online version of this article (10.1186/s11671-018-2646-7) contains supplementary material, which is available to authorized users.

## Background

The demand for rechargeable micro-power sources with compatible sizes is increasing due to the development of miniaturized electronic devices such as micro-electromechanical systems, micro-sensors, and implantable biomedical devices [1, 2]. Lithium-ion batteries, which store charges via intercalation and de-intercalation of Li ions in carbonaceous materials, have been widely used in vehicles and portable electronic devices because of their extremely high energy densities among available energy storage devices [3, 4]. However, the intrinsic aging phenomenon and instability, which are difficult to be replaced or require extremely high reliability, limit their applications [5, 6]. Electrochemical double-layer capacitor (EDLC), also known as ultra-capacitor or supercapacitor, which stores charge in the electrochemical double layer at the electrode-electrolyte interface, is a promising alternate energy storage device that possesses long lifetime and high stability [7, 8]. Unlike battery electrodes, suffering from relatively slow chemical reactions and/or severe volumetric expansion during the charge-discharge cycles, EDLCs can be operated at extremely high cycling rates because they are not limited by the relatively sluggish charge transfer kinetics between electrodes and electrolyte, leading to extremely high power densities [9]. Since electrodes of EDLC are generally composed of materials with extremely high specific surface area (SSA), their specific capacitance can be drastically increased [10].

Silicon (Si), the second most abundant element in earth, has been widely used in electronics and solar industries due to its low price and well-developed application knowledge. To achieve maximized SSA, a wide variety of methods have been proposed to fabricate silicon nanostructures using top-down or bottom-up approaches, for example, vapor-liquid-sold (VLS) deposition, reactive ion etching (RIE), electrochemical etching, or metal-assisted chemical etching [11–14]. Among these techniques, electrochemical etching is chosen to synthesize porous Si (PSi) under an atmospheric and low-temperature environment with controllable thicknesses and porosities through the etching current and the duration. However, compared with pristine doped wafers, the porous-structured electrode suffers from poor electric conductivities, largely due to surface traps [15] and deteriorated stability because of its high reactivity caused by enlarged surface area [16]. These shortcomings affect the charges inducible in the electrochemical double layers and limit the lifetime of the PSi-based EDLC. Therefore, protection of the electrode and enhancement of its conductivity are required to improve the capacitive performances of the PSi-based EDLCs. Two-dimensional structured graphene, a carbon analogue with sp^2^ hybridization, possesses excellent electronic and physiochemical properties and chemical stability as well as exceptional structural strength, which are extremely favorable to enhance electrochemical performances such as high capacities, energy densities, fast charge-discharge rates, and long lifetime for energy storage devices [17, 18]. However, a conventional transfer technique of the graphene layer cannot achieve the uniform coating on surface of nanostructures with a higher aspect ratio.

In spite of the advantages of EDLCs, the energy stored is currently lower than that of batteries by one to two orders of magnitudes, which limits their adoption to those applications that require high energy densities [19]. In theory, the higher SSA of the EDLC electrode, the more energy storage is possible within a fixed volume or weight. SSA up to 3100 m^2^/g has been achieved by creating extremely small pores [20], ranging from 1 to 10 nm, on the surface of graphene, which is called the activation of graphene. The graphene activation process is suggested to precede as 6KOH + C ⇌ 2 K + 3H_2_ + 2K_2_CO_3_. The decomposition and reaction of K_2_CO_3_/K_2_ with carbon results in pore formation [21]. In this regard, we demonstrate a uniform and conformal coating of graphene on the surface of a porous silicon matrix with excellent conductivity using a Ni vapor-assisted chemical vapor deposition (CVD) process. The interplay between the coating of the graphene and the porous structures of the PSi at different annealing temperatures can be explored to the benefit of the electrode design. Because of the highly enhanced sensitivity of the PSi structure, the pores tend to collapse at temperatures much lower than the melting point of the bulk silicon, simultaneously leading to reorganization and passivation of the electrode. The rate performance, capacitance retention, and cyclic stability of the PSi-based EDLCs fabricated from different electrode designs were then reported and investigated. The hybrid porous PSi electrode, optimized in terms of capacitive performance, achieves a high areal capacitance of 6.21 mF/cm^2^ at an ultra-high scan rate of 1000 mV/s and an unusual progressing cyclic stability of 131% at 10,000 cycles. In addition to mesopores and macropores, micropores were introduced onto the surfaces of the passivating few-layer graphene sheets with a KOH activation process to further increase the functioning surface areas of the hierarchical PSi electrode, leading to subsequent enhancement of the areal capacitance by 31.4% up to 8.16 mF/cm^2^. The present designed hierarchical PSi-based supercapacitor is proved to be a robust energy storage device for microelectronic applications that require stable high rate capability.

## Methods/Experimental

### Electrochemical Etching of Porous Silicon

Firstly, a p + doped silicon wafer kept in close contact with a titanium plate acted as the anode while a platinum electrode was used as the cathode. Then, etching solution was prepared by mixing hydrofluoric acid and dehydrated alcohol with a volume ratio of 1:1. A current density of 1 mA/cm^2^ was applied for 10 min to form a layer of etched porous structure on the pristine wafer. The wafer was then cut into a 2 × 1 cm^2^ size for subsequent experiments.

### Synthesis of PSi-based Electrodes via Ni-Assisted CVD Process

Ni ingots of 99.99% purity were placed in a Al_2_O_3_ crucible in the quartz tube, along with the as-etched porous silicon on a quartz crucible at the other end of the tube, with which precursor methane gas of ~ 50 sccm was incorporated. Oxidation of Ni ingots during the heat treatment was prevented by the creation of a reducing atmosphere with the forming gas, consisting of Ar/H_2_ of 100/20 sccm. The graphene layer can be directly grown on the PSi structure at heating temperatures of 1000~1100 °C under an increased pressure of 60 Torr.

### Characterizations

A field emission scanning electron microscope (FE-SEM, JSM-6500F, JEOL), operating at 15 kV, and a field emission transmission electron microscope (FE-TEM, JEM-3000F, JEOL) equipped with an energy dispersion spectrometer (EDS), operating at 300 kV, were used to study surface morphologies and microstructures. Raman microscope (Horiba Jobin Yvon LabRam HR800 with excitation wavelength of 632.8 nm) was employed to characterize graphene coating.

## Results and Discussion

**Fig. 1:**
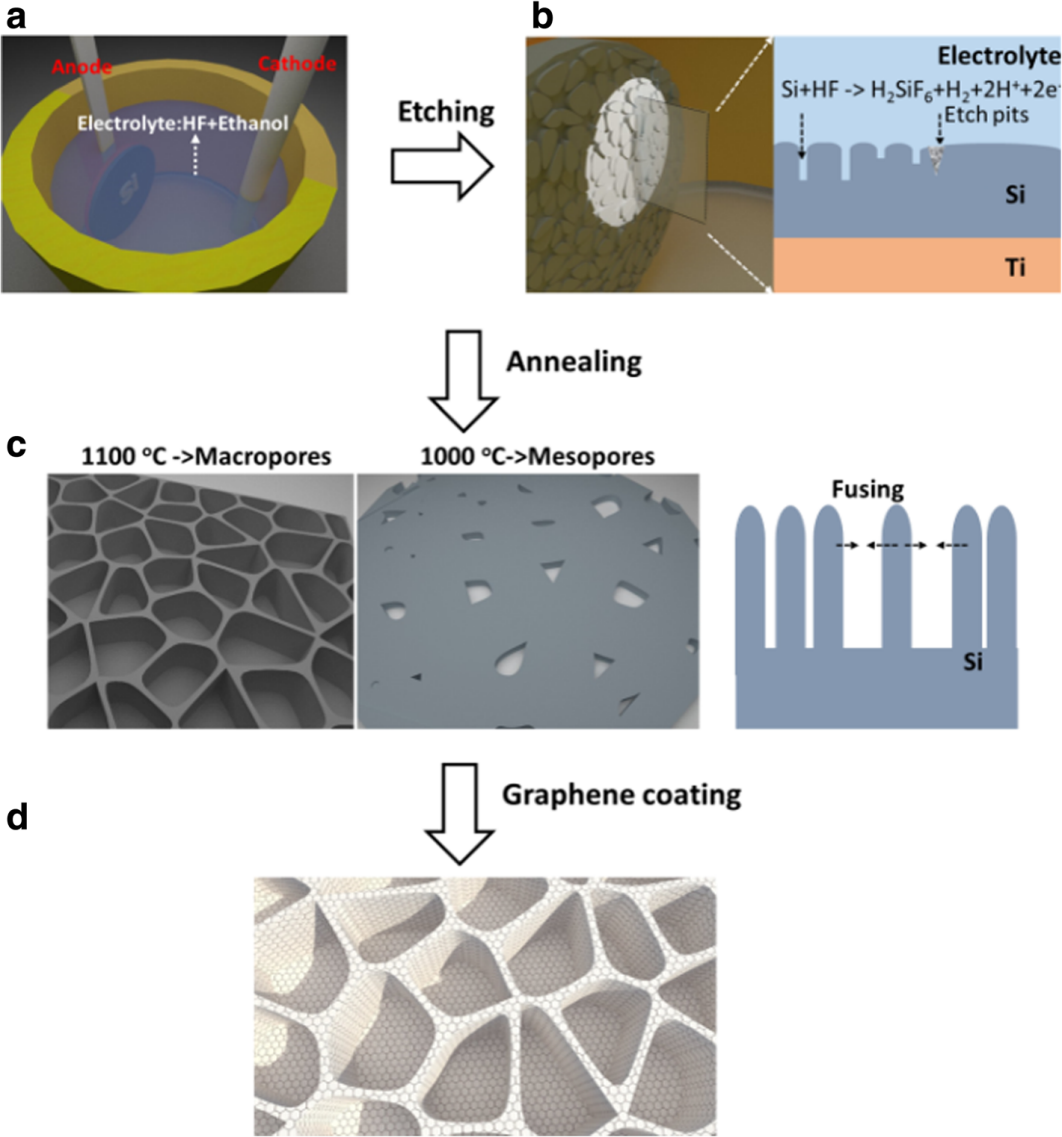
(a) and (b) Electrochemical etching of porous silicon. (c) and (d) Schematic of Ni-assisted CVD process for graphene coating and pore reorganization

Figure 1 shows overall processes from electrochemical etching of porous silicon to the Ni-assisted CVD process for graphene coating and pore reorganization [22]. Initially, pores are formed on the surface of the silicon by the electrochemical reaction of HF with Si, and further growth only occurs at the pore tips where the abundantly available holes result in the faster dissolution of silicon (Fig. 1 (a), (b)). The application of proper bias between cathode and anode has the advantageous effect to the formation of porous silicon through its dissolution by electrochemical etching process. Uniform coating of graphene sheets on the surface of such a porous silicon matrix was achieved, with conductivities and thicknesses readily controllable with growth conditions (Fig. 1 (c)). Because of the highly enhanced sensitivity of the PSi structure, the pores tend to collapse at temperatures much lower than the melting point of the bulk silicon [23], simultaneously bringing in reorganization and passivation of the electrode (Fig. 1 (c), (d)).

**Fig. 2:**
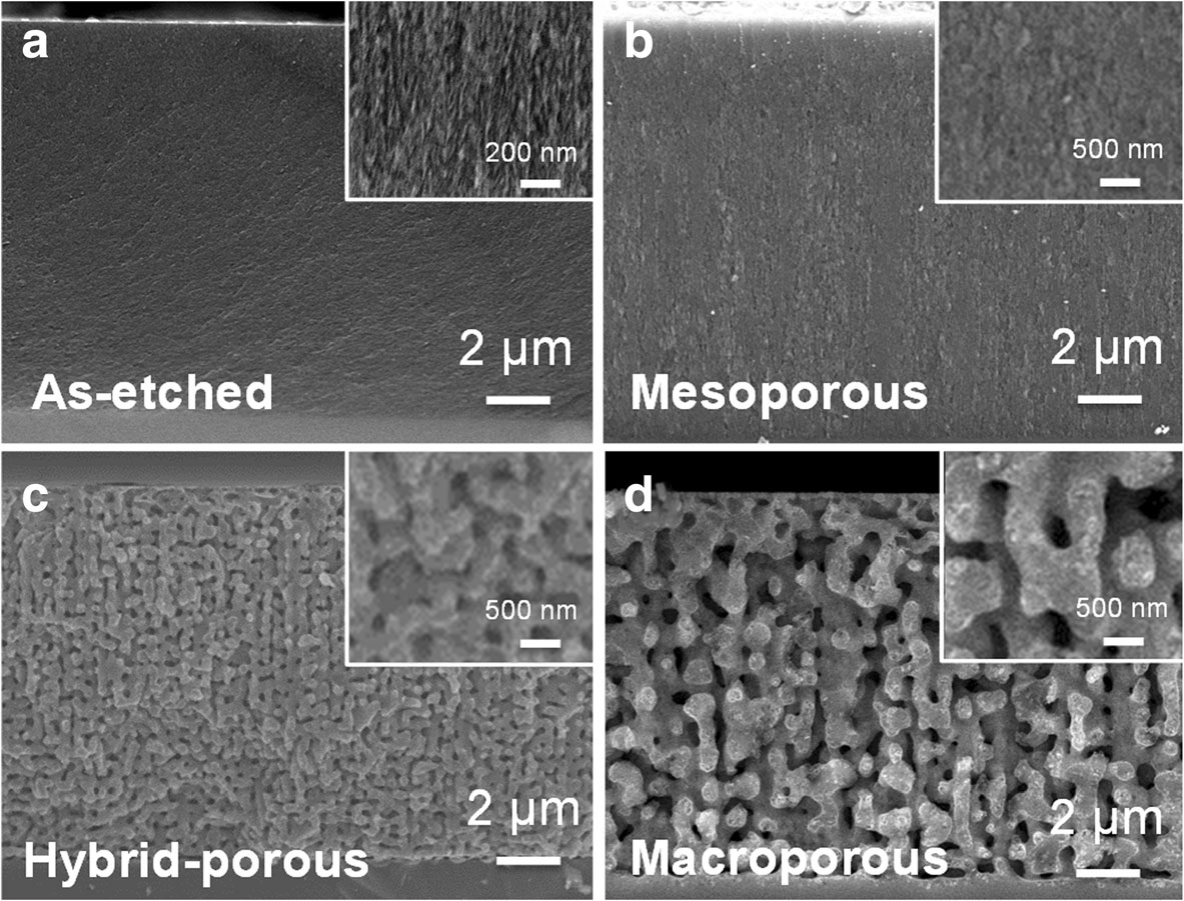
**a** Cross-sectional SEM image of as-etched PSi. **b**–**d** Cross-sectional SEM images of PSi structures after annealing at 1000, 1050, and 1100 °C. Insets show magnified images

The morphologies of the PSi matrix before and after annealing at increasing temperatures (1000, 1050, and 1100 **°**C) were shown in Fig. 2a–d for comparison. Insets in Fig. 2 show the corresponding surface morphologies. In Fig. 2a, the thickness of the as-etched Si is ~ 15 μm and the porous structure is more clearly observed. After annealing at 1000 **°**C, only a slight change in morphology took place, which is barely identifiable from the cross-section-view SEM image even with the magnification of 20,000 (Fig. 2b). The further high-magnified top-view SEM image shows that the pores are uniformly distributed in the etched region (Additional file 1: Figure S1a) where the average diameter of the pores is ~ 11 nm with the maximum size less than 20 nm that is in the range of mesopores (2~50 nm). As the annealing temperature increases to 1050 **°**C, some mesopores fuse together, forming pores with diameters greater than 50 nm and thus resulting in the hybrid porous structure composed of mesopores and macropores (> 50 nm) as shown in Fig. 2c (Additional file 1: Figure S1b). Further increase in the annealing temperature to 1100 **°**C causes the pore coalescence to an even greater extent, and all mesopores are fused to form even larger macropores as evident from Fig. 2d (Additional file 1: Figure S1c). Formation of larger macroscopic pores by the pore reorganization at such a high annealing temperature might occur to reduce the surface energy. The results present a clear idea of the precise control of pore size with the variation in different annealing temperatures.

**Fig. 3:**
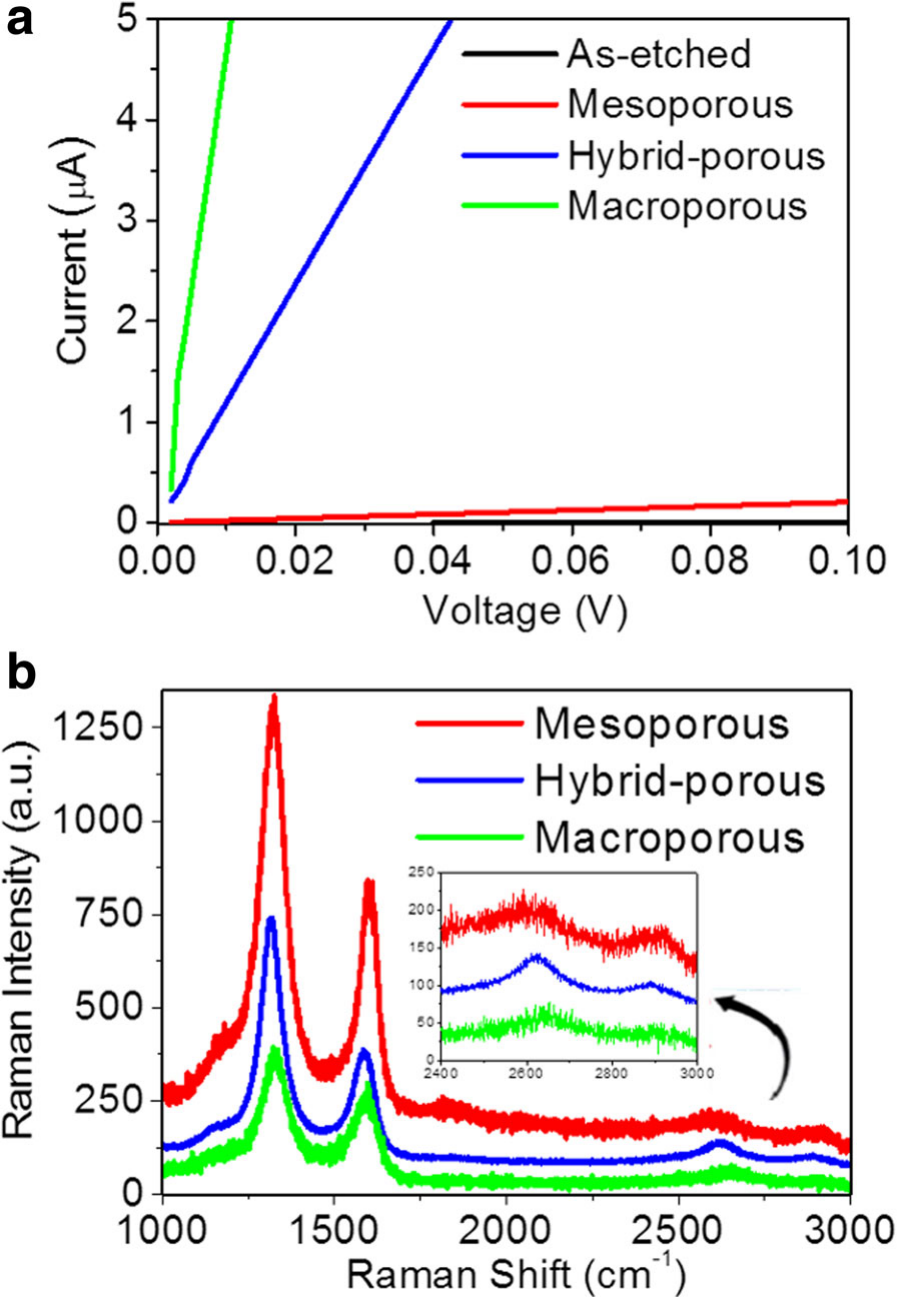
**a** I-V curves of as-etched and annealed PSis. **b** Raman spectra showing G, D, and 2D peaks of annealed PSis

Hereinafter, for better understanding of the characteristic performances of the PSi-based EDLCs fabricated at different annealing temperatures, the porous structures are denoted by their characteristic pore sizes like mesoporous, hybrid porous, and macroporous, instead of the annealing temperatures. In addition, the annealing process also provides the coating of a few-layer graphene sheet on the surfaces of the porous structures, offering their excellent conductivities to effectively reduce the resistances of the porous structures. The corresponding I-V curves with different porosities are shown in Fig. 3a. Obviously, the resistance of the porous PSi as electrode before annealing is approximately 3.3 × 10^7^ Ω and is drastically decreased by four orders of magnitude to 5.2 × 10^3^ Ω after annealing at 1000 °C. The resistance was further lowered to 85 and 22 Ω after annealing at even higher temperatures of 1050 and 1100 °C, respectively. The coating of a few-layer graphene sheet can be also confirmed by Raman spectra as shown in Fig. 3b where characteristic D band (~ 1350 cm^−1^), G band (~ 1580 cm^−1^), and 2D band (~ 2700 cm^−1^) of graphitic materials were confirmed [24]. Besides, the intensity of the D band decreases as the annealing temperature increases, indicating that defects or boundaries existing in the graphene sheets were annihilated by the healing provided by the annealing process. However, the I_2D_/I_G_ ratio, a reflection of the layer of the graphene coating, increases when the annealing temperature increases to 1050 °C but decreases at 1100 °C, revealing the existence of an optimum annealing temperature. Thus, the corresponding PSi structure containing the most well-crystalized graphene sheets is the hybrid porous PSi. In the same time, the graphene-coated PSi can have much higher chemical stability.

**Fig. 4:**
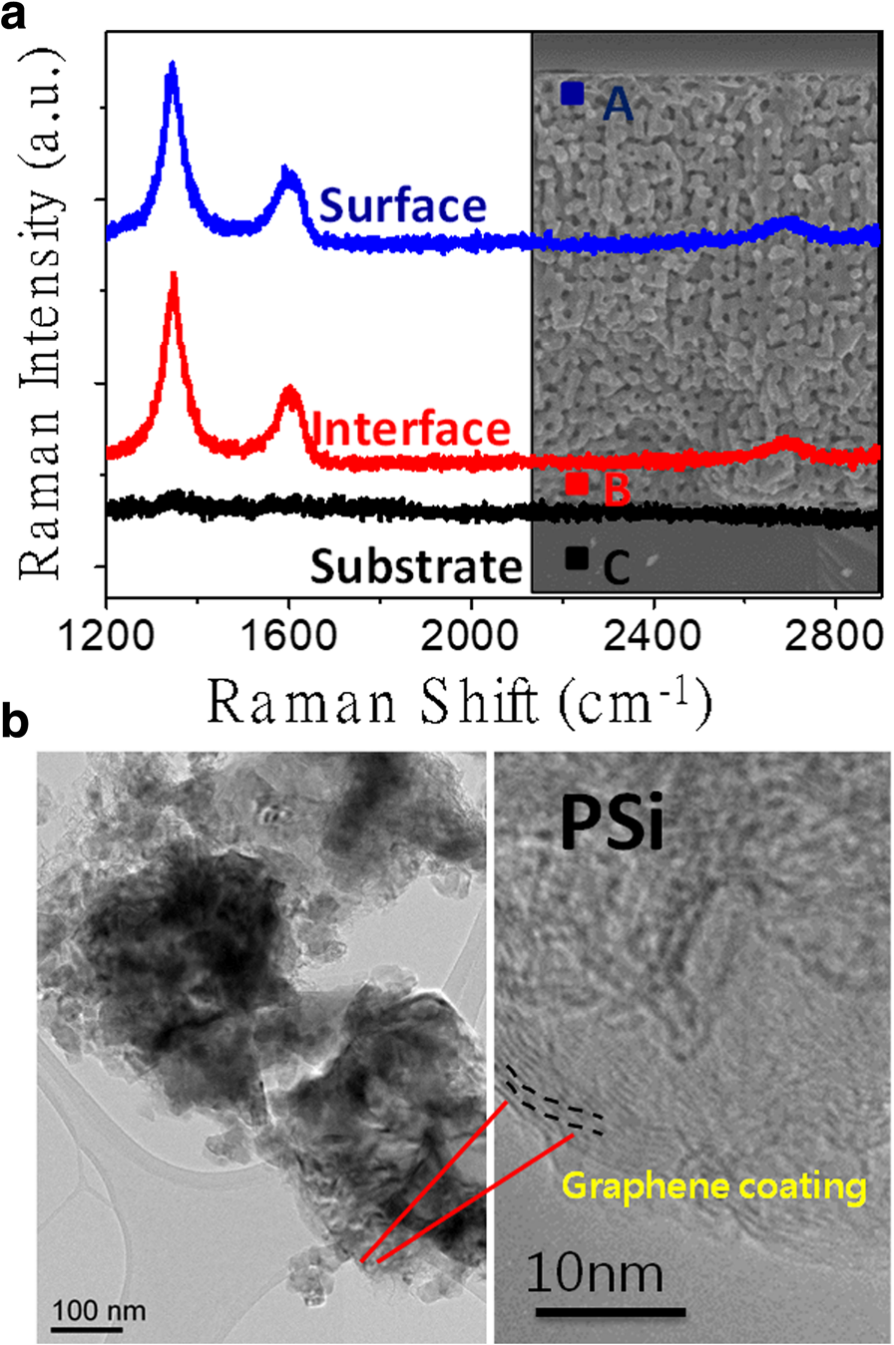
**a** Cross-sectional Raman spectra of hybrid porous PSi. **b** TEM and HR-TEM images (noticeable graphene coating) of hybrid porous PSi

To achieve full utilization of the etched porous region, the uniform coverage of the graphene coating on the PSi surfaces across the entire PSi matrix is imperative. To shed light on this part, the cross-sectional Raman spectra of the hybrid porous PSi were recorded at three representative spots, namely, point A near the structure surface, point B at the interface between the etched region and un-etched wafer, and point C in the un-etched wafer, respectively, which are shown in Fig. 4a. To our expectation, points A and B show almost the same Raman spectra while point C does not show any significant scattering peaks. This confirms that the deposition of the graphene sheets was uniform throughout the PSi matrix. The left panel of Fig. 4b is the TEM image of the hybrid porous sample, in which the darker region represents the PSi surrounded by the lighter region of graphene. The HRTEM image in the right panel of Fig. 4b shows that the hybrid PSi is in fact covered by graphene of ~ 10 layers. Furthermore, the specific capacitance was determined from a relationship of *C* = *i* (d*V*/d*t*) where *i* is the current density and d*V*/d*t* is the scan rate (V/s). The cyclic voltammetry (CV) was conducted with a potential window of 0 to 0.8 V in an environmentally benign neutral electrolyte of 0.5 M aqueous Na_2_SO_4_ solution.

**Fig. 5:**
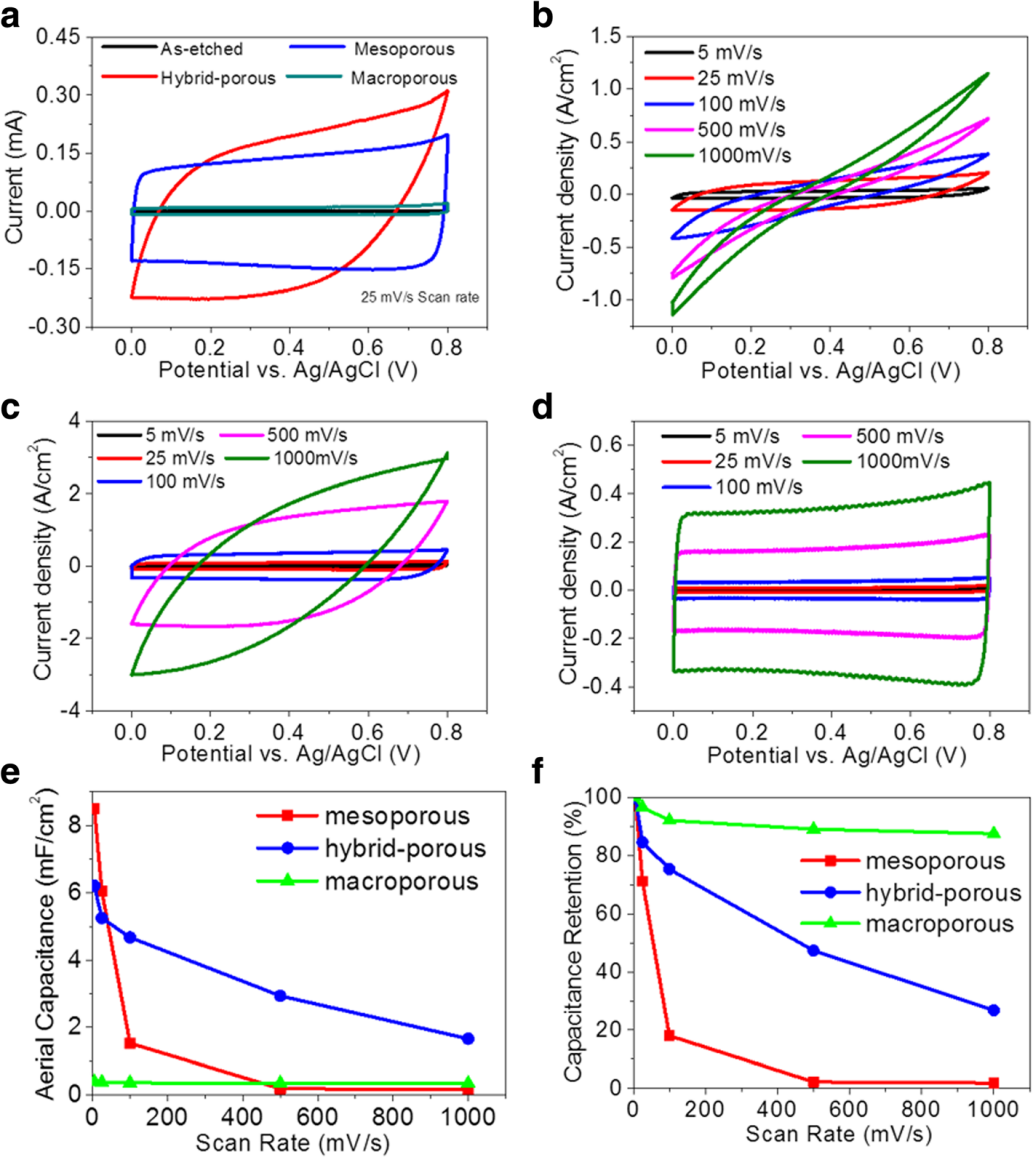
CV curves of **a** as-etched and annealed PSi electrodes at a 25 mV/s scan rate. **b** Mesoporous, **c** hybrid-porous, and **d** macroporous PSi electrodes at 5–1000 mV/s scan rates, respectively. **e** Areal capacitance and **f** capacitive retention of annealed PSi electrodes with scan rates ranging from 5 to 1000 mV/s

Figure 5a shows the CV curves of the PSi electrodes fabricated with different porosities at a scan rate of 25 mV/s. The pronounced increase in current near 0.8 V indicates the oxidation tendency of the PSi electrode. The electrodes, containing mesopores, have higher SSA and thus higher activity as well. These electrodes are more likely to be oxidized and thus show more pronounced oxidation peaks. For the hybrid porous and macroporous electrodes, the oxidation peaks are less pronounced, which may be contributed to the lower surface areas and thicker graphene coatings, both advantageous for lowering the surface activity. Still, the peak near 0.8 V may be due to the oxidation of graphene or the electrolyte at higher potential. The capacitive currents increase by two to three orders of magnitude after the annealing process, which proves that the graphene coating effectively passivates the PSi electrode and enhances conductivity for generation of the electrochemical double-layer capacitance. The shapes of the CV curves also differ with the characteristic structures of the electrodes. The preferred rectangular shape measured from the macroporous PSi electrode indicates that larger pore sizes reduce the mass transfer resistance within the porous structure and improve the accessibility of the electrolyte to the pore surfaces for capacitance generation. Figure 5b–d compares the CV curves of the PSi electrodes with different porous structures at scan rate ranges of 5–1000 mV/s. At lower scan rates, despite the lower conductance, the mesoporous PSi electrode exhibits the higher capacitive current due to its higher SSA. The CV curves of all the electrodes show a rectangular shape at the scan rate of 5 mV/s. However, as the scan rate increases, the CV curves of the mesoporous and hybrid porous PSi electrodes show progressive degree of inclination while that of the macroporous PSi electrode barely changes. It is because of the presence of the large pores that helps improve the accessibility of the electrolyte to the surfaces of the pores. In fact, the capacitance results from the accumulation of charge on the pore surface, which is related to the process of the electrolyte penetration through the porous structure and formation of the electric double layer. Thus, the electrolyte can easily diffuse through the paths of the large pores while the diffusion time through smaller pores is long. The areal specific capacitance and the high rate retention of the PSi electrodes of different porous structures at different scan rates are shown in Fig. 5e, f. The areal capacitance is computed using the following equation:

$$ \mathrm{CA}\kern0.5em =\frac{\int iV\mathrm{d}v}{2\mu A\Delta V} $$

where *i* and *V* are the generated capacitive current and applied potential in the CV measurement, *μ* is the scan rate (V/s), *A* is the apparent area of the electrode, and Δ*V* is the working potential window (0.8 V in this case), respectively. As a result, the mesoporous PSi electrode shows the highest areal capacitance of 8.48 mF/cm^2^ at the scan rate of 5 mV/s but only 0.1% retention at the scan rate of 1000 mV/s. On the contrary, the areal capacitance of the macroporous PSi electrode is only 0.396 mF/cm^2^ at the scan rate of 5 mV/s, but with an excellent retention of 87.5% at the scan rate of 1000 mV/s. As for the hybrid-porous PSi electrode, it shows a decent specific capacitance of 6.21 mF/cm^2^ while maintaining a satisfactory retention of 47.3% at a higher scan rate of 500 mV/s, suitable for common or extreme purposes. Thus, the hybrid porous PSi electrode was chosen for further investigation.

**Fig. 6:**
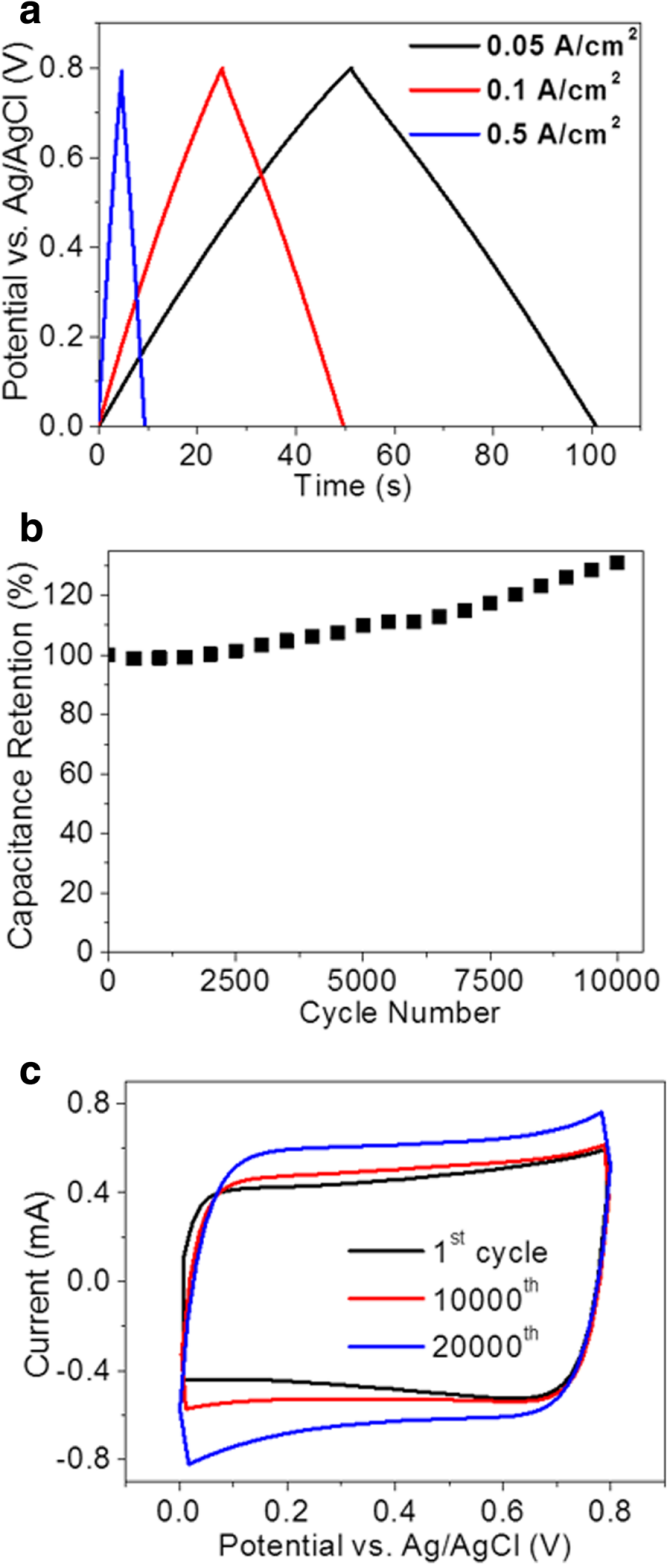
**a** Galvanostatic charge-discharge curves of the hybrid porous PSi electrode. **b** Capacitance retention of hybrid porous PSi electrode over 10,000 cycles. **c** CV curves of hybrid porous PSi electrode at 1st, 10,000th, and 20,000th cycles

**Fig. 7:**
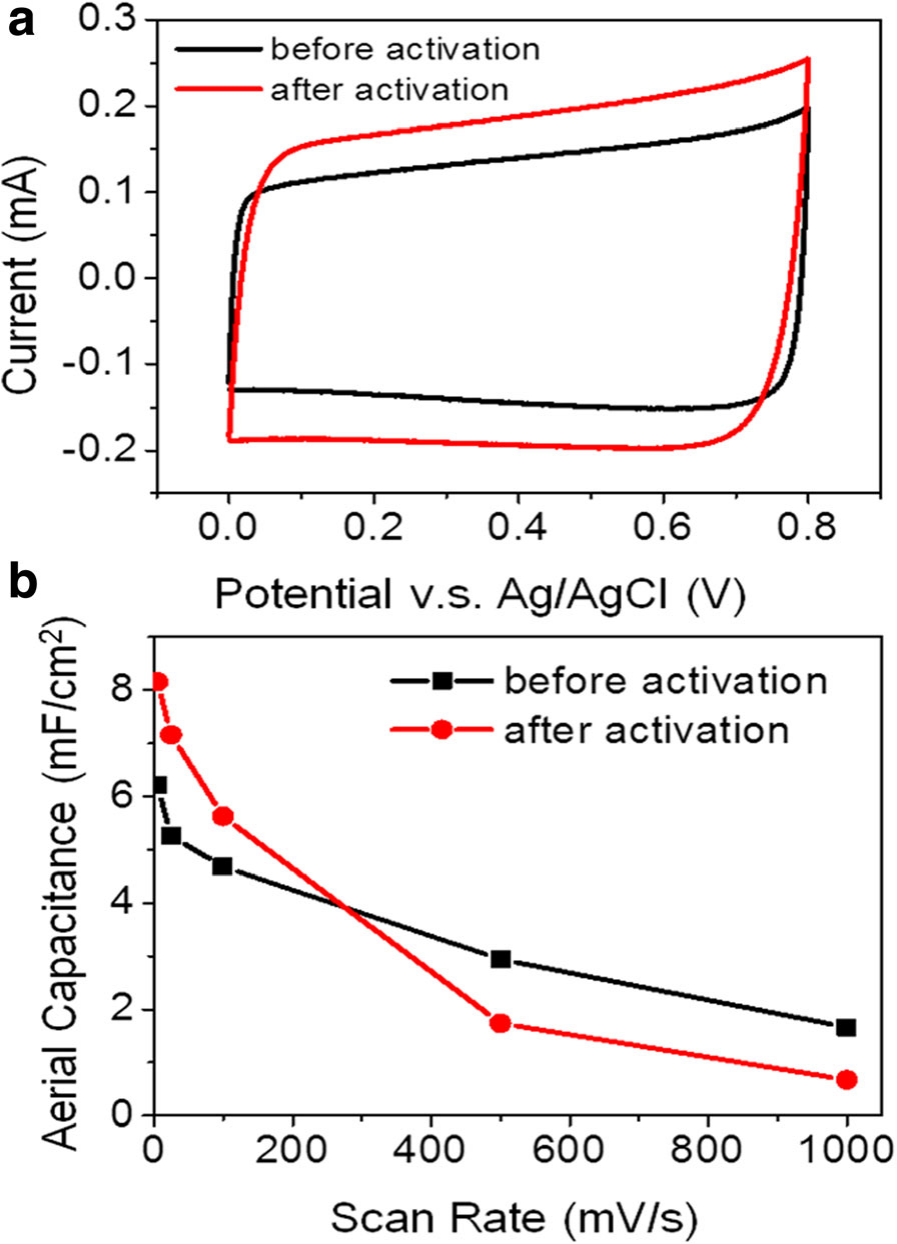
**a** C-V curves of the hybrid porous PSi electrode before and after activation at 100 mV/s. **b** The variation in areal capacitance of hybrid porous PSi electrodes before and after activation with the identical scan rate

In Fig. 6a, the charge-discharge curves of the hybrid porous PSi electrode are consistently triangular at increasing current densities with a columbic efficiency over 90%. The stability of the device was demonstrated in Fig. 6b where an increase of 31% in the areal capacitance was observed over 10,000 cycles at the scan rate of 100 mV/s. This may be attributed to the improvement in the wettability of the electrolyte to the electrode, resulting from the oxidation of the electrode material discussed above. Figure 6c shows the CV curves of the hybrid porous PSi electrode recorded at the 1st, 10,000th, and 20,000th cycles, respectively, all exhibiting the preferred rectangular shape. Apart from mesopores and macropores, the introduction of micropores (< 5 nm) will increase the SSA of the PSi electrode which accounts for the further enhancement in its capacitive performances. To introduce micropores onto the graphene coating, the electrode was dipped in a 3.5-M KOH basic solution for 1 min and then baked in vacuum at 800 °C for 30 min with a 200-sccm argon flow. The areal capacitance at relatively lower scan rates, e.g., 5 mV/s, was pumped up by 31.4% from 6.21 to 8.16 mF/cm^2^ with the KOH activation process while a decrease in the areal capacitance was observed when the scan rate gets higher than 200 mV/s as shown in Fig. 7a, b. In addition to the case of mesopores, micropores also give rise to much higher mass transfer resistances for the electrolyte to penetrate through it. Consequently, longer times are needed for the formation of the electrochemical double layers and the electrode cannot respond to the scan in time. Nevertheless, the areal capacitance at scan rate lower than 100 mV/s was effectively enhanced by the activation process. A comparison of this work with other Si-based supercapacitors in terms of areal capacitance and cycling stability is summarized in Table 1. Though there is a lag in the areal capacitance value of synthesized supercapacitor material in the present case with respect to some of the existing materials, the demonstration of its excellent long-term cycling stability shows the great competitiveness of this work with the available active materials [25–29].

**Table 1:** Comparative study of the current work with the available literatures in terms of long-term cycling stability and areal capacitance

Type of material	Passivation (material/process)	Capacitance retention (retention/cycle number)	Areal capacitance (mF/cm^2^)	Reference
SiNWs	N/A	99.5%/200	0.046	[25]
SiNWs	SiC/CVD	95%/1000	1.7	[26]
SiC NWs	N/A	95%/200,000	0.24	[27]
PSi	Graphene/CVD	94.9%/5000	N/A	[28]
PSiNWs	Graphene/CVD	83%/5000	325	[29]
PSi	Graphene/CVD	131%/10,000	8.19	This work

## Conclusions

The PSi-based supercapacitors with different porous structures have been realized by electrochemical etching of silicon wafer and subsequent passivation with graphene coating via the CVD process. The capacitive performances of the PSi EDLCs are closely related to the composition of the porous structure consisting of macropores, mesopores, and/or micropores. The present activated hybrid porous PSi electrode operates in an environmentally benign aqueous solution and exhibits high specific capacitances, excellent cycling stability, and satisfactory high rate retention at an extremely high scan rate of 1000 mV/s. The capacitive performances were further boosted via an activation process that effectively increases the areal capacitance to be comparably higher among other Si-based EDLCs.

## Additional File

Additional file 1: **Figure S1.** (a)~(c) Top-view SEM images of PSi structures after annealing at 1000, 1050, and 1100 °C. (DOCX 243 kb)
