# Supplementary material for: Few-Layer Graphene Sheet-Passivated Porous Silicon Toward Excellent Electrochemical Double-Layer Supercapacitor Electrode
Source: Nanoscale Res Lett. 2018 Aug 17;13:242. doi: 10.1186/s11671-018-2646-7 (PMC6097977; doi:10.1186/s11671-018-2646-7)
Supplement: Supplementary file 1 — Figure S1. (a)~(c) Top-view SEM images of PSi structures after annealing at 1000, 1050, and 1100 °C. (DOCX 243 kb) [file 11671_2018_2646_MOESM1_ESM.docx]

**Supplementary Information**

**Few-layer Graphene Sheets Passivated Porous Silicon Toward Excellent Electrochemical Double Layer Supercapacitor Electrode**

Te-Hui Wu,^a, c, d+^ Chih-Tse Chang, ^a, c, d+^ Chun-Chieh Wang,^b+^ Shaikh Parwaiz, ^a, c, d^ Chih-Chung Lai, ^a, c, d^ Yu-Ze Chen, ^a, c, d^ Shih-Yuan Lu^b*^ and Yu-Lun Chueh,^a, c, d*^

*^a^ Department of Materials Science and Engineering, National Tsing Hua University, Hsinchu 30013, Taiwan.*

*^b^Department of Chemical Engineering, National Tsing-Hua University, Hsinchu 30013, Taiwan, ROC.*

*^c^ Department of Physics, National Sun Yat-Sen University, Kaohsiung, 80424, Taiwan, ROC.*

*^d^Frontier Research Center on Fundamental and Applied Sciences of Matters, National Tsing Hua University, Hsinchu 30013, Taiwan.*

Te-Hui Wu ^a+^

E-mail: tehuiwu@gmail.com

Chih-Tse Chang^a++^

E-mail: ctchang1991@hotmail.com

Chun-Chieh Wang^b+^

E-mail: brian628628@gmail.com

Shaikh Parwaiz^a^

E-mail: parwaiz.shaikh1993@gmail.com

Chih-Chung Lai^a^

E-mail: neilonlai@gmail.com, lai.cc@nsrrc.org.tw

Yu-Ze Chen^a^

E-mail: kinorassic@gmail.com

Shih-Yuan Lu^b*^

E-mail: sylu@mx.nthu.edu.tw

Yu-Lun Chueh^a,c*^

E-mail: ylchueh@mx.nthu.edu.tw

E-mail: ylchueh@mx.nthu.edu.tw and sylu@mx.nthu.edu.tw


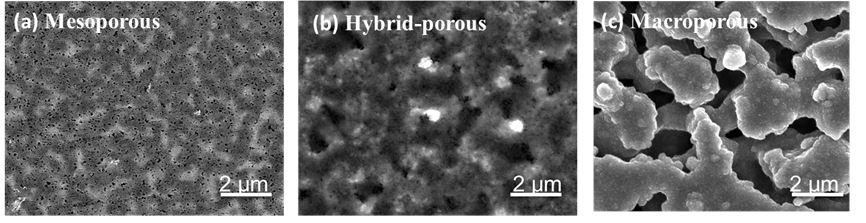


**Figure S1.** (a)~(c) Top view SEM images of PSi structures after annealing at 1000, 1050 and 1100 **^◦^**C
